# BrevicidineB, a New Member of the Brevicidine Family, Displays an Extended Target Specificity

**DOI:** 10.3389/fmicb.2021.693117

**Published:** 2021-06-09

**Authors:** Xinghong Zhao, Oscar P. Kuipers

**Affiliations:** Department of Molecular Genetics, Groningen Biomolecular Sciences and Biotechnology Institute, University of Groningen, Groningen, Netherlands

**Keywords:** antimicrobial activity, Brevicidine, NRPs, cyclic peptide, lipopeptide

## Abstract

The group of bacterial non-ribosomally produced peptides (NRPs) has formed a rich source for drug development. Brevicidine, a bacterial non-ribosomally produced cyclic lipo-dodecapeptide, displays selective antimicrobial activity against Gram-negative pathogens. Here, we show that brevicidineB, which contains a single substitution (Tyr2 to Phe2) in the amino acid sequence of the linear part of brevicidine, has a broadened antimicrobial spectrum, showing bactericidal activity against both Gram-negative (with a MIC value of 2 to 4 mg/L) and Gram-positive (with a MIC value of 2 to 8 mg/L) pathogens. Compared with an earlier reported member of the brevicidine family, the broadened antimicrobial spectrum of brevicidineB is caused by its increased membrane disruptive capacity on Gram-positive pathogens, which was evidenced by fluorescence microscopy assays. In addition, DiSC3(5) and resazurin assays show that brevicidine and brevicidineB exert their antimicrobial activity against Gram-negative bacteria via disrupting the proton motive force of cells. Notably, as a brevicidine family member, brevicidineB also showed neither hemolytic activity nor cytotoxicity at a high concentration of 64 mg/L. This study provides a promising antibiotic candidate (brevicidineB) with a broad antimicrobial spectrum, and provides novel insights into the antimicrobial mode of action of brevicidines.

## Graphical Abstract

The antimicrobial mode of action of brevicidines. Brevicidines are potent antimicrobial cyclic lipopeptides active against Gram-negative pathogens. They have low hemolytic activity and cytotoxicity. BrevicidineB, a single amino acid residue mutant of brevicidine, displays an altered antimicrobial spectrum, showing also antimicrobial activity against Gram-positive pathogens. Due to disruption of the proton motive force of Gram-negative pathogens, brevicidines act as bactericidal antibiotics. In addition, brevicidineB acts as a bactericidal antibiotic against Gram-positive pathogens, because of its disruption capacity of the cellular membrane.

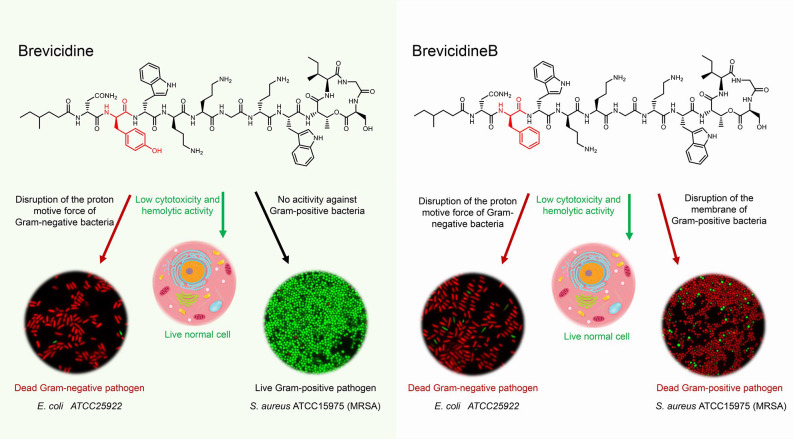

## Introduction

Brevicidine (Bre), a cyclic lipopeptide, was discovered from *Brevibacillus laterosporus* DSM 25 by genome mining (Li et al., 2018). This cyclic lipopeptide displays selective antimicrobial activity against Gram-negative pathogens, including *Enterobacter cloacae*, *Escherichia coli*, *Pseudomonas aeruginosa*, *Klebsiella pneumoniae*, and *Acinetobacter baumannii* (Li et al., 2018). Notably, Bre has relatively low cytotoxicity and hemolytic activity, and it has been shown to display effective antimicrobial effects in mice infected with *E. coli* (Li et al., 2018). This information demonstrates that Bre could be developed as an excellent antibiotic candidate against Gram-negative pathogens. Although Bre was reported 3 years ago, little is known about how Bre exerts its selective antimicrobial activity against Gram-negative pathogenic bacteria.

In this study, brevicidineB (BreB), containing a single substitution (Tyr2 to Phe2) in the amino acid sequence of the linear part of Bre, was discovered from the producer strain of Bre, *Brevibacillus laterosporus* DSM 25. It is exciting that BreB shows antimicrobial activity against both Gram-negative and Gram-positive pathogens. Therefore, we envisioned that this broadened antimicrobial spectrum of BreB could be based on an altered antimicrobial mechanism. Subsequently, the antimicrobial mechanisms of Bre and BreB were investigated.

The results show that Bre exerts its selective antimicrobial activity against Gram-negative pathogens by disrupting the proton motive force of the cellular membrane. BreB employs the same mode of action against Gram-negative pathogens as Bre. However, due to its membrane permeabilization ability, BreB also has antimicrobial activity against Gram-positive pathogens. Finally, hemolytic activity and cytotoxicity assays show that there is no innate safety concern for BreB.

## Materials and Methods

### Bacterial Strains Used and Growth Conditions

Strains used in this study are listed in Supplementary Table 1. *Brevibacillus laterosporus* DSM 25 cells were inoculated in LB and incubated at 37°C for prepare overnight culture. For production of brevicidines, an overnight culture of *Brevibacillus laterosporus* DSM 25 cells was inoculated (50-fold dilution) in minimal expression medium (MEM) and grown 36 h at 30 ^0^C. All indicator strains were inoculated in LB and incubated at 37°C for preparing the overnight cultures.

### Purification of Cationic Cyclic Lipopeptides From *Brevibacillus laterosporus* DSM25 Culture Medium

A 36 h old *Brevibacillus laterosporus* DSM 25 culture was centrifuged at 15,000 *g* for 15 min, and the supernatant was collected and adjusted the pH to 7. After that, the culture was applied to a CM Sephadex^TM^ C-25 column (GE Healthcare) equilibrated with distilled water. The flow-through was discarded, and the column was subsequently washed with 12 column volumes (CV) of distilled water. The peptide was eluted with 6 CV 2 M NaCl. The eluted peptide was then applied to a SIGMA-ALDRICH C18 Silica gel spherically equilibrated with 10 CV of 5% aq. MeCN containing 0.1% trifluoroacetic acid. After washing with a 10 CV of 5% aq. MeCN containing 0.1% trifluoroacetic acid, peptides were eluted from the column using up to 10 CV of 50% aq. MeCN containing 0.1% trifluoroacetic acid. Fractions containing the eluted peptides were freeze-dried and dissolved in MQ water. After filtration through a 0.2 μm filter, the cyclic lipopeptides were purified on an Agilent 1260 Infinity HPLC system with a Phenomenex Aeris C18 column (250 × 4.6 mm, 3.6 μm particle size, 100 Å pore size). Acetonitrile was used as the mobile phase, and a gradient of 25-35% aq. MeCN over 30 min at 1 mL per min was used for separation. The purified lipo-tridecapeptides were eluted with a gradient of 28 to 32% aq. MeCN. The expression levels for Bre and BreB were 6 mg/L and 0.5 mg/L, respectively.

### Mass Spectrometry

Matrix-assisted laser desorption ionization-time-of-flight (MALDI-TOF) mass spectrometer analysis was performed using a 4800 Plus MALDI TOF/TOF Analyzer (Applied Biosystems) in the linear-positive mode as in previously studies (Zhao et al., 2020a,b,c). Briefly, a 1 μL sample was spotted on the target, and dried at room temperature. Subsequently, 0.6 μL of matrix solution (5 mg/mL of αα-cyano-4-hydroxycinnamic acid) was spotted on each sample. After the samples had dried, MALDI-TOF MS was performed.

### LC-MS/MS Analysis

Due to LC-MS/MS can be used to yield b and y ions for peptides, it is widely used in peptide structure elucidation (; McClerren et al., 2006; Zhang et al., 2014; Zhao et al., 2020b). To gain insight into the peptides molecular structures LC-MS/MS was performed. LC-MS was performed using a Q-Exactive mass spectrometer fitted with an Ultimate 3000 UPLC, an ACQUITY BEH C18 column (2.1 × 50 mm, 1.7 μm particle size, 200 Å; Waters), a HESI ion source and an Orbitrap detector. A gradient of 5–90% MeCN with 0.1% formic acid (v/v) at a flowrate of 0.35 mL/min over 60 min was used. MS/MS was performed in a separate run in PRM mode selecting the doubly and triply charged ion of the compound of interest.

### Minimum Inhibitory Concentration (MIC) Assays

Minimum inhibitory concentration values were determined by broth micro-dilution according to the standard guidelines (Wiegand et al., 2008). Briefly, the test medium was cation-adjusted Mueller-Hinton broth (MHB). Cell concentration was adjusted to approximately 5 × 10^5^ cells per mL. After 20 h of incubation at 37°C, the MIC was defined as the lowest concentration of antibiotic with no visible growth. Each experiment was performed in triplicate.

For measuring the MIC of antimicrobials against indicator strains in the presence of 10% human plasma, MHB was replaced with MHB containing 10% human plasma and the effect of these two conditions were compared (Li et al., 2018).

### Assay for Time-Dependent Killing

This assay was performed according to a previously described procedure (Ling et al., 2015; Zhao et al., 2020c). An overnight culture of either *Escherichia coli* ATCC25922 or *Staphylococcus aureus* ATCC15975 (MRSA) was diluted 50-fold in MHB and incubated at 37°C with aeration at 220 r.p.m. Bacteria were grown to an OD of 0.5, and then the concentration of cells was adjusted to ≈5 × 10^6^ cells per mL for *E. coli* and ≈2 × 10^7^ for *S. aureus*. Bacteria were then challenged with 10 × MIC antimicrobials in glass culture tubes at 37°C and 220 r.p.m. Bacteria not treated with peptides were used as a negative control. At desired time points, two hundred μl aliquots were taken, centrifuged at 8,000 *g* for 2 min and resuspended in 200 μl of MHB. Ten-fold serially diluted samples were plated on MHA plates. After incubation at 37°C overnight, colonies were counted and the c.f.u. per mL was calculated. Each experiment was performed in triplicate.

### Fluorescence Microscopy Assays

This assay was performed according to a previously described procedure (Li et al., 2020a,b). *Escherichia coli* ATCC25922 or *Staphylococcus aureus* ATCC15975 (MRSA) was grown to an OD_600_ of 0.8. Culture was pelleted at 4,000 g for 5 min and washed three times in MHB. After normalization of the cell density to an OD_600_ of 0.2 in MHB, brevicidines were added at final concentrations of 2 × MIC, 1 × MIC or 0.5 × MIC, while nisin/polymyxin B was added at a concentration of one-fold MIC. At the same time, SYTO 9 and propidium iodide (LIVE/DEAD Baclight Bacterial Viability Kit, Invitrogen) were added to the above cell suspensions. After incubation at room temperature for 15 min, peptides were removed and washed three times with MHB. Then the cell suspensions were loaded on 1.5% agarose pads and analyzed by DeltaVision Elite microscope (Applied precision).

### DiSC3(5) Assays

DiSC3(5) assay was adapted from previously described procedures (Li et al., 2020a; Stokes et al., 2020). Briefly, *Escherichia coli* ATCC25922 was grown to an OD_600_ of 0.8. The cell culture was pelleted at 5,000 g for 5 min and washed three times in MHB. The cell density was normalized to an OD_600_ of 0.2, loaded with 6 μM DiSC_3_(5) dye (3,3’-dipropylthiadicarbocyanine iodide), and incubated for 20 min in the dark for probe fluorescence to stabilize. Subsequently, the cell suspension was added to a 96-well microplate and incubated for 20 min. After antibiotics were added at final concentrations of 2 × MIC, 1 × MIC or 0.5 × MIC, with the antibiotics added after ∼20 s, fluorescence was monitored for 30 min. The excitation and emission wavelengths on the fluorescence spectrometer were adjusted to 622 nm and 670 nm, respectively. Representative examples from three technical replicates are shown.

### Resazurin Assay

Non-fluorescent resazurin can be reduced to resorufin, a highly fluorescent compound, in a NAD(P)H dependent manner in the presence of NAD(P)H dehydrogenase (Barnes and Spenney, 1980; Winartasaputra et al., 1980; McMillian et al., 2002; Li et al., 2020a). Therefore, the resazurin/NAD(P)H dehydrogenase/NAD(P)H system can be used to detect the NAD(P)H level of cells. *Escherichia coli* ATCC25922 cells were grown in MHB until the OD_600_ reached 0.1. Resazurin was added to the cell culture at a final concentration of 100 μg per mL. Brevicidines and polymyxin B were added at final concentrations of 2 × MIC, 1 × MIC or 0.5 × MIC. Milli Q water was used as a untreated control. Fluorescence was recorded by using a Thermo Scientific Varioskan LUX multimode microplate reader with excitation and emission wavelengths of 622 nm and 670 nm, respectively. Representative examples from three technical replicates are shown.

### Hemolytic Activity Assay

Hemolytic activity assay was performed as the method described in previous studies (Ling et al., 2015; Li et al., 2018). In short, erythrocytes were isolated from the blood of a healthy human and washed with 0.1 M PBS three times. Subsequently, peptides were added at final concentrations of 64, 32, 16, 8, 4, 2, and 0 μg/mL in PBS containing 2% (v/v) erythrocytes. The cells were incubated at 37°C for 1 h and centrifuged for 5 min at 10,000 *g*. The supernatant was transferred to a 96-well plate, and the absorbance was measured at a wavelength of 570 nm with a Thermo Scientific Varioskan LUX multimode microplate reader. The absorbance relative to the positive control, which was treated with 10% Triton X-100, was defined as the percentage of hemolysis. Representative examples from three technical replicates are shown.

### Mammalian Cytotoxicity

The cytotoxicity of brevicidines was evaluated on a human liver cell line (HepG2) cells by using the XTT (Cell Proliferation Kit XTT, AppliChem) assay (Roehm et al., 1991). HepG2 cells (in Dulbecco’s Modified Eagle’s medium supplemented with 10% Fetal Bovine Serum) were seeded into 96-well plates, and incubated at 37 °C with 5% CO_2_. After 24 h, the medium was replaced with fresh medium (DMEM with 2% FBS, 100μL per well) containing different concentrations of brevicidines (Zhao et al., 2017). After 24 h incubation, the XTT reagent was added to the cultures according to the manufacturer’s instructions, and the plates were incubated at 37°C for 2 h with 5% CO_2_. Subsequently, the absorbance values were measured by using a Varioskan LUX multimode microplate reader (Thermo Fisher Scientificı) at 485 nm (reference 690 nm).

### Quantification and Statistical Analyses

GraphPad Prism 7.0 was used to fit the data of time-killing assays, DiSC_3_(5) assays, resazurin reduction assays, hemolytic assays, and cytotoxicity assays in Figures 2, 4, 5. Experiments were conducted in triplicate, and data are represented as the mean value of triplicate experiments; Data Explorer Software was used to analyze the MALDI-TOF data; Thermo Scientific Xcalibur software was used to analyze the LC-MS/MS data; ImageJ was used to analyze the fluorescence microscopy images. The statistical significance of the data was assessed using a two-tailed Student’s t-test with GraphPad Prism 7.0. Correlation analyses were evaluated by Pearson r2, ns: *p* > 0.05, ^∗^*p* < 0.05, ^∗∗^*p* < 0.01, and ^∗∗∗^*p* < 0.001.

## Results

### Isolation and Characterization of Purified Cyclic Lipopeptides by MALDI-TOF MS and LC-MS/MS

**FIGURE 1:**
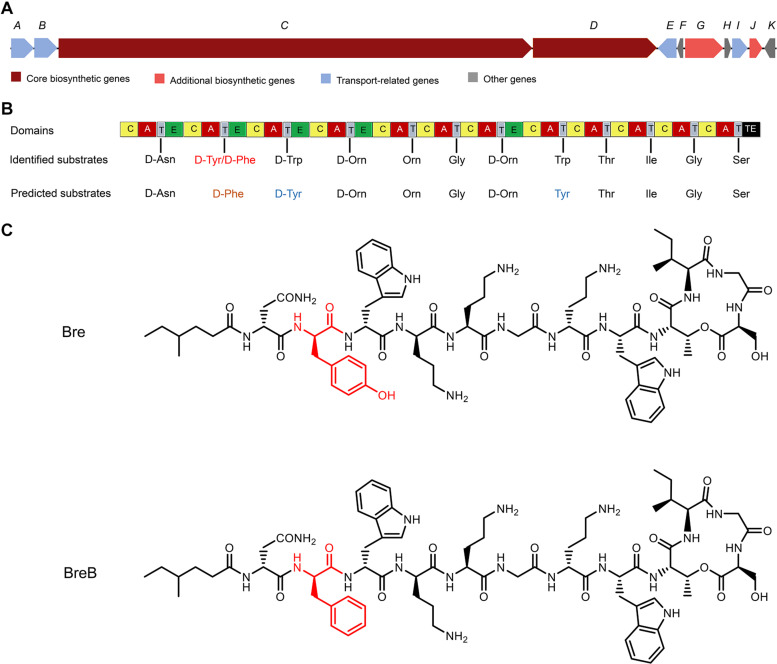
The structures of brevicidines and the predicted biosynthetic gene cluster. **(A)** The non-ribosomal peptide synthetases genes harbored by the *Brevibacillus laterosporus* DSM 25 genome. **(B)** the catalytic domains encoded by the gene cluster, and the substrates incorporated by the respective modules. Domains: A, adenylation; T, thiolation; C, condensation; E, epimerization; TE, thioesterase. **(C)** structures of brevicidines. The red color indicates the different structure of brevicidines.

As the structure of Bre is different from the peptide that was revealed by the antimicrobial mining software antiSMASH version 5.1.1 (Medema et al., 2011; Blin et al., 2019; Figures 1A, B), we envisioned that *Brevibacillus laterosporus* DSM 25 could produce some variants of Bre since most of the NRP biosynthetic systems are able to produce several natural analogs. In the present study, cationic peptides were purified from *Brevibacillus laterosporus* DSM 25 using the method described in a previous study (Zhao et al., 2020b). After HPLC purification (Supplementary Figure 1), MALDI-TOF MS was used to measure molecular weight of the purified peptides. The two purified compounds were analyzed by MALDI-TOF MS and their masses were determined to be 1519 Da (compound **1**) and 1503 Da (compound **2**) (Supplementary Figures 2, 3), respectively. Further characterizations were performed by an LC-MS/MS analysis, and the results are shown in Supplementary Figures 4, 5. The structures of these two compounds were elucidated by using known Bre as a reference template. Compound **1** is the reported cyclic lipopeptide, Bre, and compound **2** is a novel cyclic lipopeptide, termed BreB, which has a single substitution (Tyr2 to Phe2) in the amino acid sequence of the linear part of Bre (Figure 1C).

**FIGURE 2:**
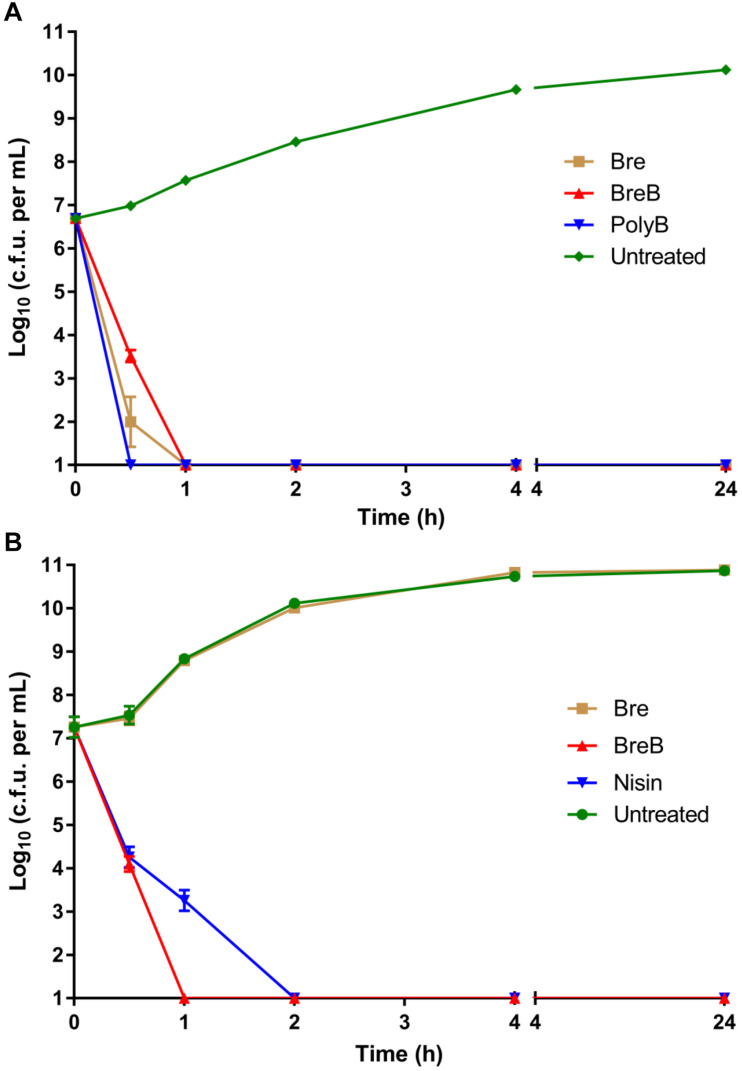
BrevicidineB acts as a bactericidal antibiotic against both Gram-negative and Gram-positive pathogenic bacteria. **(A)** time killing curve of brevicidines (10 × MIC) against *Escherichia coli*. Each experiment was performed in triplicate. **(B)** time killing curve of brevicidines (10 × MIC) against *Staphylococcus aureus* (MRSA). Each experiment was performed in triplicate.

**FIGURE 3:**
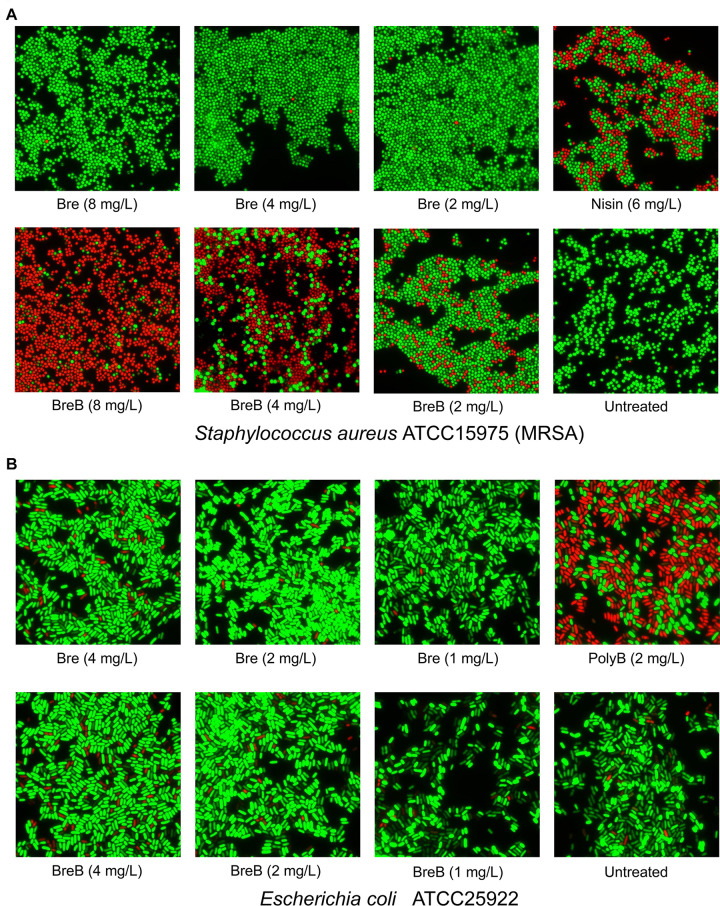
BrevicidineB acts as a potent antibiotic against Gram-positive bacteria by disruption of the cellular membrane. **(A)** Fluorescence microscopy image of *Staphylococcus aureus* cells after being exposed to nisin (6 mg/L; 1 × MIC), brevicidine (8 mg/L, 4 mg/L, or 2 mg/L), and brevicidineB [8 mg/L (2 × MIC), 4 mg/L (1 × MIC) or 2 mg/L (0.5 × MIC)] for 15 min. **(B)** Fluorescence microscopy image of *E. coli* cells after being exposed to polymyxin B (2 mg/L; 1 × MIC), brevicidine [4 mg/L (2 × MIC), 2 mg/L (1 × MIC) or 1 mg/L (0.5 × MIC)], and brevicidineB [4 mg/L (2 × MIC), 2 mg/L (1 × MIC), or 1 mg/L (0.5 × MIC)] for 15 min. Green denotes a cell with an intact membrane, whereas red denotes a cell with a compromised membrane.

**FIGURE 4:**
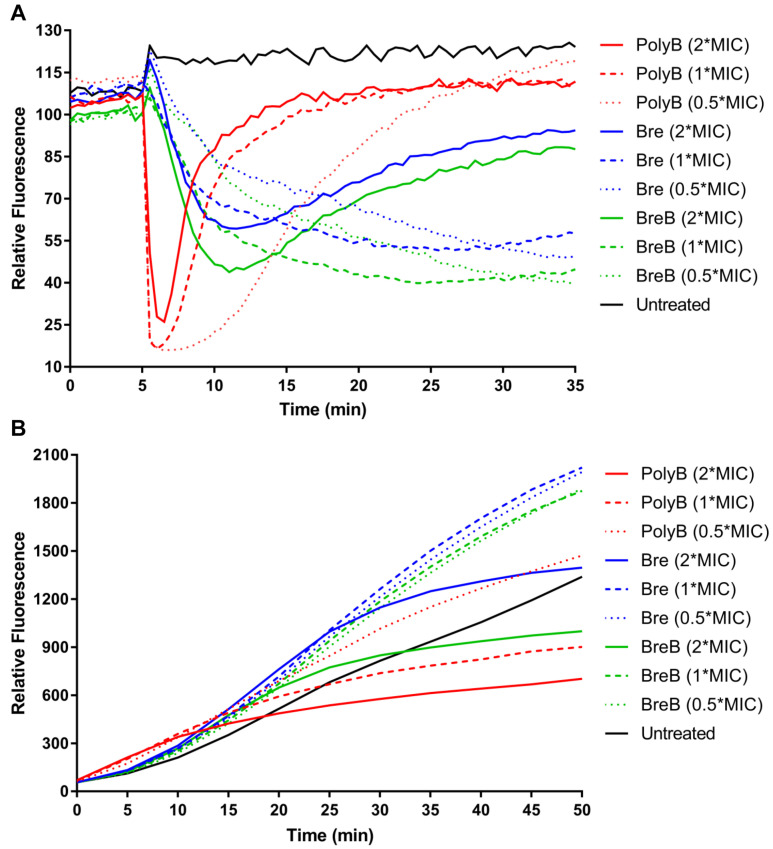
Brevicidines disrupt the proton motive force of Gram-negative bacteria. **(A)** DiSC_3_(5) fluorescence in *E. coli* upon exposure to polymyxin B [4 mg/L (2 × MIC), 2 mg/L (1 × MIC) or 1 mg/L (0.5 × MIC)], Bre [4 mg/L (2 × MIC), 2 mg/L (1 × MIC) or 1 mg/L (0.5 × MIC)], and BreB [4 mg/L (2 × MIC), 2 mg/L (1 × MIC) or 1 mg/L (0.5 × MIC)]. Representative examples from three technical replicates are shown. **(B)** Resorufin fluorescence in *E. coli* upon exposure to polymyxin B [4 mg/L (2 × MIC), 2 mg/L (1 × MIC) or 1 mg/L (0.5 × MIC)], Bre [4 mg/L (2 × MIC), 2 mg/L (1 × MIC) or 1 mg/L (0.5 × MIC)], and BreB [4 mg/L (2 × MIC), 2 mg/L (1 × MIC) or 1 mg/L (0.5 × MIC)]. Representative examples from three technical replicates are shown.

**FIGURE 5:**
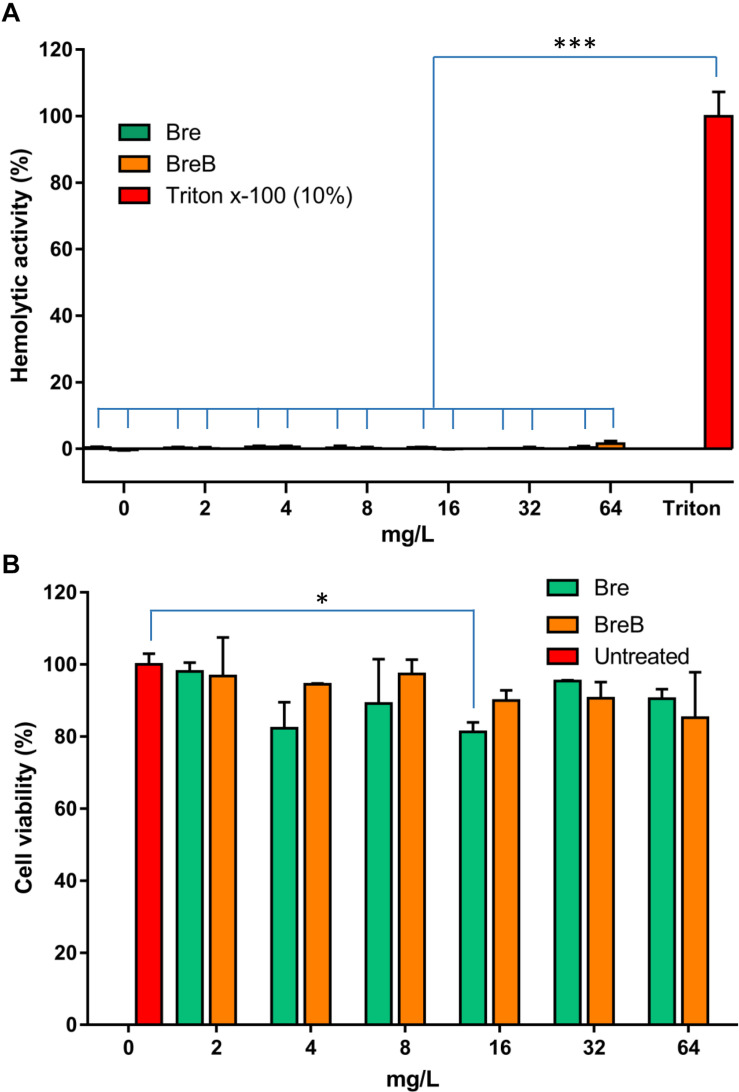
Brevicidines show exceptional low cytotoxicity and hemolytic activity. **(A)** Human erythrocytes were incubated with brevicidines at concentrations ranging from 2 to 64 mg/L. Their hemolytic activity was assessed by the release of hemoglobin. Cells treated without a tested compound were used as no-lysis control. Cells treated with 10% Triton X-100 were used as complete lysis control. The data are representative of three independent experiments. Correlation analyses were evaluated by Pearson r2, ns: *p* > 0.05, *p < 0.05, and ***p < 0.001 vs. 10% Triton X-100-treated cells. **(B)** Cytotoxicity of brevibacillins against HepG2 cells. The data are representative of three independent experiments. Correlation analyses were evaluated by Pearson r2, ns: *p* > 0.05, *p < 0.05, and ***p < 0.001 vs. Untreated cells.

### BreB, a Single Amino Acid Residue Mutant of Bre, Displays a Broadened Antimicrobial Spectrum

The antimicrobial activity of brevicidines against pathogenic bacteria was measured by MIC assays. Polymyxin B and nisin were used as antibiotic controls. Nisin showed good antimicrobial activity against the Gram-positive pathogens tested, but it had insufficient antimicrobial activity against the Gram-negative pathogens tested. Polymyxin B showed strong antimicrobial activity against most of the tested Gram-negative pathogens; however, polymyxin B resistance was observed in *Enterobacter cloacae* LMG02783. Moreover, polymyxin B showed insufficient antimicrobial activity against the tested Gram-positive pathogens. In agreement with a previous study, Bre showed strong antimicrobial activity against Gram-negative pathogens (Table 1; Li et al., 2018). The newly discovered member of the Bre family, BreB, also showed good antimicrobial activity against all the tested Gram-negative pathogens, including *Enterobacter cloacae*, *Escherichia coli*, *Pseudomonas aeruginosa*, *Klebsiella pneumoniae*, and *Acinetobacter baumannii*, with a MIC value of 2 to 4 mg/L (Table 1). These results demonstrate that BreB has similar antimicrobial activity as Bre against Gram-negative pathogens. Subsequently, the antimicrobial activity of Bre and BreB against Gram-positive pathogens was tested. In agreement with a previous study (Li et al., 2018), Bre showed no antimicrobial activity against the tested Gram-positive pathogens (Table 1). Interestingly, BreB showed good antimicrobial activity against all the tested Gram-positive pathogens, including difficult-to-treat antibiotic-resistant *Enterococcus* and *Staphylococcus aureus* (Table 1), with a MIC value of 2 to 8 mg/L. These results suggest that BreB is a better antibiotic candidate than Bre, polymyxin B, or nisin in treating mixed infections since it has good antimicrobial activity against both Gram-positive and Gram-negative pathogens. BreB showed the same antimicrobial activity in the presence or absence 10% human blood plasma against Gram-positive pathogens [*S. aureus* ATCC15975 (MRSA) or *E. faecium* LMG16003]. Interestingly, brevicidines and polymyxin B showed much stronger antimicrobial activity against *Escherichia coli*, *Pseudomonas aeruginosa* (PAO1), *Klebsiella pneumoniae* in the presence of 10% human blood plasma (Table 1), which may relate to their effective antimicrobial activity *in vivo* (Li et al., 2018). The enhanced antimicrobial activity of brevicidines in the presence of human blood plasma makes them more attractive for further development as antimicrobials.

**TABLE 1:** MIC values of Bre and BreB against pathogenic bacteria.

Microorganism	MIC (mg/L)			
	
	Bre	BreB	PolyB	Nisin
**Gram-negative pathogenic bacteria**				
*Enterobacter cloacae* LMG02783	1	2	>64	32
*Escherichia coli* ATCC25922	2	2	2	64
*Escherichia coli* ATCC25922^*a*^	0.25	0.25	0.5	N.D.
*Pseudomonas aeruginosa* PAO1	2	2	1	>64
*Pseudomonas aeruginosa* PAO1^*a*^	0.5	1	0.25	N.D.
*Klebsiella pneumoniae* LMG20218	2	4	2	>64
*Klebsiella pneumoniae* LMG20218^*a*^	0.25	1	0.5	N.D.
*Pseudomonas aeruginosa* LMG6395	4	4	2	>64
*Acinetobacter baumannii* ATCC17978	16	4	4	32
**Gram-positive pathogenic bacteria**				
*Enterococcus faecium* LMG16003 (VRE)	>64	2	16	6
*Enterococcus faecium* LMG16003 (VRE)^*a*^	>64	2	N.D.	6
*Staphylococcus aureus* ATCC15975 (MRSA)	>64	4	32	6
*Staphylococcus aureus* ATCC15975 (MRSA)^*a*^	>64	4	N.D.	6
*Bacillus cereus* ATCC14579	>64	4	8	6
*Enterococcus faecalis* LMG16216 (VRE)	>64	8	16	6

*^*a*^MIC values tested in MHB plus 10% Human plasma. VRE, vancomycin resistant *Enterococcus*; MRSA, methicillin resistant *S. aureus*; polyB is polymyxin B. N.D., not determined.*

### BreB Acts as a Bactericidal Antimicrobial Against Both Gram-Negative and Gram-Positive Pathogens

Investigating the time dependence of antibiotic action is widely used to identify whether a compound is bacteriostatic or bactericidal (Ling et al., 2015; Zhao et al., 2020c). In this study, we monitored the killing kinetics of brevicidines against *E. coli* and *S. aureus*. Polymyxin B and nisin were used as bactericidal antibiotic controls for Gram-negative and Gram-positive pathogens, respectively. Polymyxin B showed the fastest killing capacity against *E. coli*, which killed all *E. coli* cells in half an hour. Brevicidines also showed a quick killing capacity against *E. coli*, which killed all the *E. coli* cells in 1 h at a desirable concentration at the site of infection (10 × MIC) (Figure 2A). BreB showed bactericidal activity against *S. aureus*, and it killed all the *S. aureus* cells in 1 h at a desirable concentration at the site of infection (10 × MIC) (Figure 2B), and has an even faster killing ability than the well-known bactericidal antimicrobial nisin (Figure 2B). However, a normal growth curve was observed for the *S. aureus* cells treated with Bre (Figure 2B). These results demonstrate that Bre acts as a bactericidal antimicrobial against *E. coli*, and BreB acts as a bactericidal antimicrobial against both *E. coli* and *S. aureus*.

### BreB Exerts Its Antimicrobial Activity Against Gram-Positive Bacteria by Disrupting the Cellular Membrane

As BreB showed antimicrobial activity against Gram-positive pathogens, we monitored the effect of brevicidines on the membrane permeability of Gram-positive bacteria. To this end, a fluorescence microscopy assay was performed on *S. aureus* cells in MHB using a commercial LIVE/DEAD Baclight Bacterial Viability Kit, which consists of SYTO 9 and propidium iodide. Cells with an intact membrane will stain green, whereas cells with a compromised membrane will stain red. Nisin was used as a membrane permeability disruptive antibiotic control. After treatment with nisin at a concentration of 6 mg/L (1 × MIC) for 15 min, around half of the *S. aureus* cells were showing in red, which demonstrates that nisin permeabilized the membrane of these red cells (Figure 3A). Interestingly, after treatment with BreB at a concentration of 4 mg/L (1 × MIC) for 15 min, more than half of the *S. aureus* cells were showing in red, and the percentage of the red cells is positively related to the concentration of BreB (Figure 3A). However, green cells were observed for the Bre (at the same concentrations used for BreB) treated *S. aureus* cells (Figure 3A). These results demonstrate that BreB disrupted the membrane of *S. aureus* cells, whereas Bre showed no influence on the membrane permeability of *S. aureus* cells (Figure 3A). These results suggest that BreB exerts its antimicrobial activity against Gram-positive bacteria by disrupting the cellular membrane, which can explain why a single amino acid mutation can broaden the antimicrobial spectrum of Bre.

### BreB Disrupts the Proton Motive Force of Gram-Negative Bacteria

To monitor the effect of brevicidines on the membrane permeability of Gram-negative bacteria, we performed a membrane permeability assay on *E. coli* cells using the same method described above. After a 2 mg/L (1 × MIC) concentration of polymyxin B treatment for 15 min, more than half of the *E. coli* cells were stained red (Figure 3B), which demonstrates that polymyxin B disrupted the cellular membrane of these red *E. coli* cells. In contrast, brevicidines showed no influence on the membrane permeability of *E. coli* cells at concentrations of 2 mg/L (1 × MIC) and 1 mg/L (0.5 × MIC), which showed stained green *E. coli* cells as the untreated group (Figure 3B). In addition, brevicidines caused membrane permeabilization for a few percent of *E. coli* cells at a concentration of 4 mg/L (2 × MIC) (Figure 3B), which suggests that brevicidines might non-specifically cause membrane permeabilization at a relatively high concentration, as many other antimicrobial peptides do (Oren and Shai, 1998; Huang et al., 2004; Brogden, 2005; Varney et al., 2013; Bakhtiary et al., 2017). These results indicate that brevicidines do not exert their antimicrobial activity against Gram-negative bacteria by increasing the membrane permeability of bacteria at low MIC value concentrations.

The dye DiSC_3_(5) (3,3’-dipropylthiadicarbocyanine iodide) is widely used in the mode of action studies of antibiotics (Rink et al., 2007; Jangra et al., 2019; Valderrama et al., 2019; Stokes et al., 2020). DiSC_3_(5) accumulates in the cytoplasmic membrane in response to the Δψ component of the proton motive force and self-quenches its fluorescence (Wu et al., 1999; Stokes et al., 2020). When ΔpH is disrupted, cells compensate by increasing the Δψ, resulting in enhanced DiSC_3_(5) uptake into the cytoplasmic membrane and therefore decreased fluorescence (Wu et al., 1999; Stokes et al., 2020). In this study, DiSC_3_(5) assays were performed in MHB by using *E. coli* cells. The fluorescence was immediately decreased after *E. coli* cells were exposed to polymyxin B, and the fluorescence then increased quickly (Figure 4A). These results demonstrate that polymyxin B disrupted the membrane proton motive force immediately after it was added to *E. coli* cells, and then fluorescence increased quickly due to polymyxin B disrupting the cellular membrane. Brevicidines showed fast but slower membrane proton motive force disruption capacity than polymyxin B in a dose-dependent manner (Figure 4A). Brevicidines showed no membrane permeabilization at concentrations of 2 mg/L (1 × MIC) and 1 mg/L (0.5 × MIC) during 30 min monitoring (Figure 4A). In agreement with the fluorescence microscopy results (Figure 3B), brevicidines (4 mg/L; 2 × MIC) started to disrupt the membrane of cells after 7 min treatment due to their non-specific membrane permeabilization capacity at a relatively high concentration, as many other antimicrobial peptides do (Figure 4A; Oren and Shai, 1998; Huang et al., 2004; Brogden, 2005; Varney et al., 2013; Bakhtiary et al., 2017). These results demonstrate that brevicidines exert their antimicrobial activity against Gram-negative pathogens by disrupting the proton motive force of the bacterial cells at low MIC value concentrations.

To further confirm that brevicidines disrupt the proton motive force of Gram-negative bacteria, the NADH level of cells was monitored by using the non-fluorescent dye resazurin, which is reduced to resorufin, a highly fluorescent compound, by NADH in the presence of NADH dehydrogenase (Barnes and Spenney, 1980; Winartasaputra et al., 1980; McMillian et al., 2002; Li et al., 2020a). Therefore, the fluorescence signal level can reflect the NADH level of cells. The fluorescence signal of antibiotic-treated cells was rapidly increasing during 10 min after treatment (Figure 4B), indicating polymyxin B, Bre, and BreB treatment increase NADH level of cells. After 15 min treatment, polymyxin B blocked the reduction of resazurin, which is consistent with the above results that polymyxin B causes immediate proton motive force disruption and thereafter disrupts the membrane and kills the bacteria (Figures 4A,B). The NADH levels of *E. coli* cells treated with brevicidines at concentrations of 2 mg/L (1 × MIC) and 1 mg/L (0.5 × MIC) were much higher than those of the untreated cells during 50 min monitoring (Figure 4B). Brevicidines blocked the reduction of resazurin after 25 min treatment at a concentration of 4 mg/L (2 × MIC), which is consistent with the DiSC_3_(5) assay results that show that brevicidines started to disrupt the membrane after 7 min of treatment due to their non-specific membrane permeabilization capacity at this relatively high concentration (Figures 4A,B). These results indicate that brevicidines disrupt the electron transport chain, which is responsible for the proton motive force of cells.

### BreB Shows Low Cytotoxicity and Hemolytic Activity

To assess the safety of brevicidines to human beings or animals in an initial test, the hemolytic activity of brevicidines to human red blood cells and the cytotoxicity of brevicidines to a human liver cell line (HepG2) were monitored. For the hemolytic activity assay, human blood cells were incubated in the presence of concentrations of brevicidines ranging from 2 mg/L to 64 mg/L. After 1 h incubation, the OD_450_ of the supernatants was monitored, and the hemolytic activities of the brevicidines were calculated as described in the previous studies (Ling et al., 2015; Li et al., 2018). Both BreB and Bre showed low hemolytic activity, showing no hemolytic activity at a relatively high concentration of 64 mg/L (Figure 5A). For cytotoxicity assays, HepG2 cells were incubated in the presence of concentrations of brevicidines ranging from 2 mg/L to 64 mg/L. After 24 h incubation, the number of viable cells was determined using an XTT kit. Both BreB and Bre showed low cytotoxicity, showing no cytotoxicity at the relatively high concentration of 64 mg/L (Figure 5B). These results are consistent with a previous study that showed Bre had low cytotoxicity and hemolytic activity (Li et al., 2018). These results do not indicate any *a priori* safety concerns for brevicidines.

## Discussion

Bre has been shown to exert selective antimicrobial activity against Gram-negative bacteria (Li et al., 2018). Unlike Bre, the newly discovered natural analog of Bre, BreB, also shows antimicrobial activity against Gram-positive pathogens due to its membrane disruption capacity on Gram-positive bacteria. The Gram-positive bacterial membrane permeabilization capacity of BreB is most likely caused by the presence of Phe2, instead of Tyr2 of Bre (Eisenberg, 1984). This Phe2 mutation makes the N-terminal part of BreB more hydrophobic than Bre and makes it able to disrupt the membrane of Gram-positive bacteria. These findings provide novel insights to develop new antibiotics based on brevicidines.

Bre has shown good antimicrobial activity both *in vitro* and *in vivo*, and it has shown a low risk of resistance development (Li et al., 2018). However, little is known about its antimicrobial mode of action, even though its activity was reported 3 years ago. In the present study, we show that Bre and the newly discovered BreB exert their antimicrobial activity against Gram-negative pathogens by disrupting the proton motive force of bacterial cells. This was further confirmed by a resazurin reduction assay, which showed that brevicidines disrupt the electron transport chain of the tested Gram-negative pathogens. Proton motive force is essential for bacteria to generate adenosine triphosphate (ATP), a process that is essential for cell growth (Bakker and Mangerich, 1981; Ahmed and Booth, 1983). Cochrane et al. reported that the antimicrobial lipopeptide tridecaptin A1 selectively kills Gram-negative bacteria by disrupting the proton motive force of bacterial cells (Cochrane et al., 2016). Moreover, nigericin, subtilosin, and valinomycin have been reported to exert their antimicrobial activity by disrupting the proton motive force of bacterial cells (Ahmed and Booth, 1983; Molenaar et al., 1991; Noll et al., 2011). These results provide new insights into the antimicrobial mode of action of Bre and BreB.

Natural products from plants, animals, and microorganisms represent a treasury for drug discovery. Nature-derived products and natural product mimics still constitute an important source for therapeutic drugs. For instance, 32 of the 132 drugs approved by the FDA in 2008-2012 are natural product mimics or are derived from natural products (Tao et al., 2015). Here, the discovered BreB and the earlier reported Bre could be used as templates for the development of optimized Bre-derived antibiotics, i.e., a novel strategy has been reported recently, which uses a ribosomal synthesis and post-translational modification way to mimic the structure and function of Bre (Zhao et al., 2020b). In addition, the total chemical synthesis of Bre was recently reported in *ChemRxiv* (Al-Ayed et al., 2021), which will contribute to further structure-activity studies.

In summary, our results show that the here-discovered novel cyclic lipopeptide, BreB, has antimicrobial activity against both Gram-negative and Gram-positive pathogens. Compared to Bre, the broadened antimicrobial spectrum of BreB is caused by its increased membrane disruption capacity on the tested Gram-positive pathogens. In addition, our mode of action studies show that Bre and BreB exert their antimicrobial activity against Gram-negative bacteria by disrupting the proton motive force of cells. This study provides novel insights into the antimicrobial mode of action of Bre and BreB, and points to a promising antibiotic candidate (BreB) with a broad antimicrobial spectrum.

## Data Availability Statement

The original contributions presented in the study are included in the article/Supplementary Material, further inquiries can be directed to the corresponding author.

## Author Contributions

OK and XZ conceived the project and strategy. XZ designed and carried out the experiments, analyzed data, and wrote the manuscript. OK supervised the work and corrected the manuscript. Both authors commented on the manuscript text and approved its final version.

## Conflict of Interest

The authors declare that the research was conducted in the absence of any commercial or financial relationships that could be construed as a potential conflict of interest.
