# Supplementary material for: BrevicidineB, a New Member of the Brevicidine Family, Displays an Extended Target Specificity
Source: Front Microbiol. 2021 Jun 9;12:693117. doi: 10.3389/fmicb.2021.693117 (PMC8219939; doi:10.3389/fmicb.2021.693117)
Supplement: Supplementary file 1 [file Data_Sheet_1.DOC]

Supplementary material

BrevicidineB, a new member of the brevicidine family, displays an extended target specificity

Xinghong Zhao, Oscar P. Kuipers *

Department of Molecular Genetics, Groningen Biomolecular Sciences and Biotechnology Institute, University of Groningen, Groningen, 9747 AG, The Netherlands.

* Correspondence: o.p.kuipers@rug.nl (Oscar P. Kuipers)


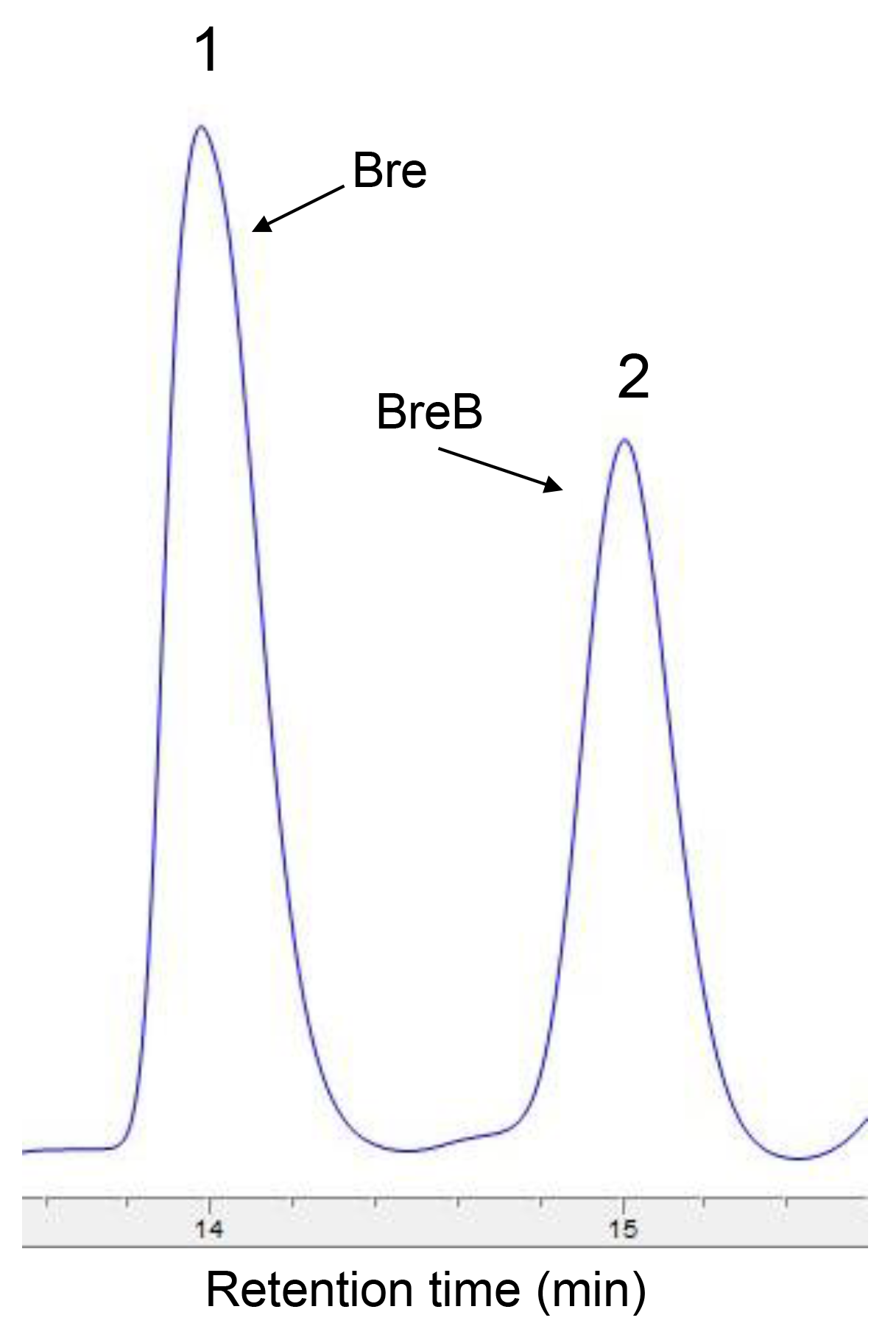


**Supplementary Figure 1.** HPLC chromatogram trace of isolated cyclic lipopeptide antibiotics; Compound 1 was identified as Bre, and compound 2 was identified as BreB by following studies.


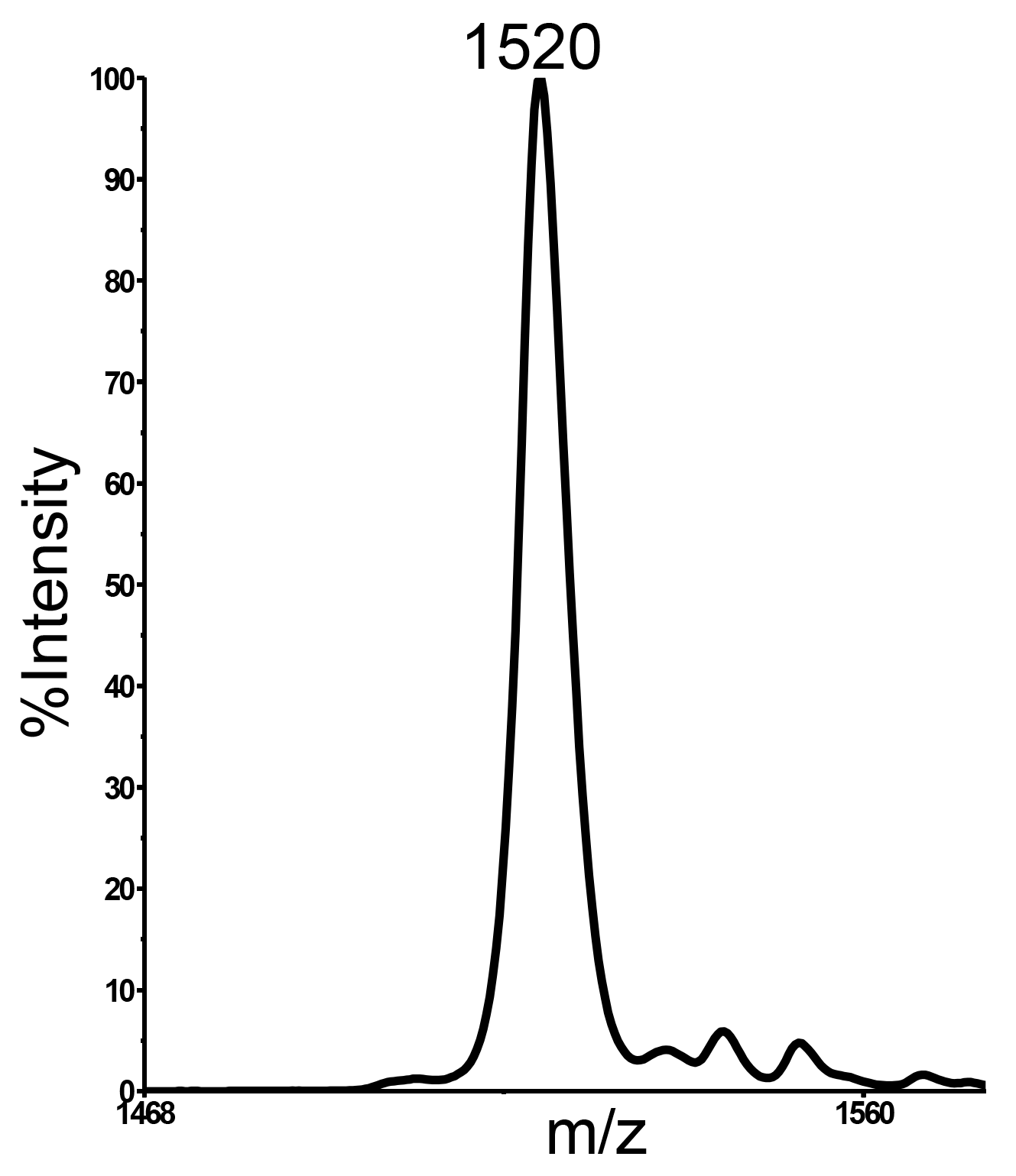


**Supplementary Figure 2.** MALDI-TOF MS data of HPLC purified Bre.


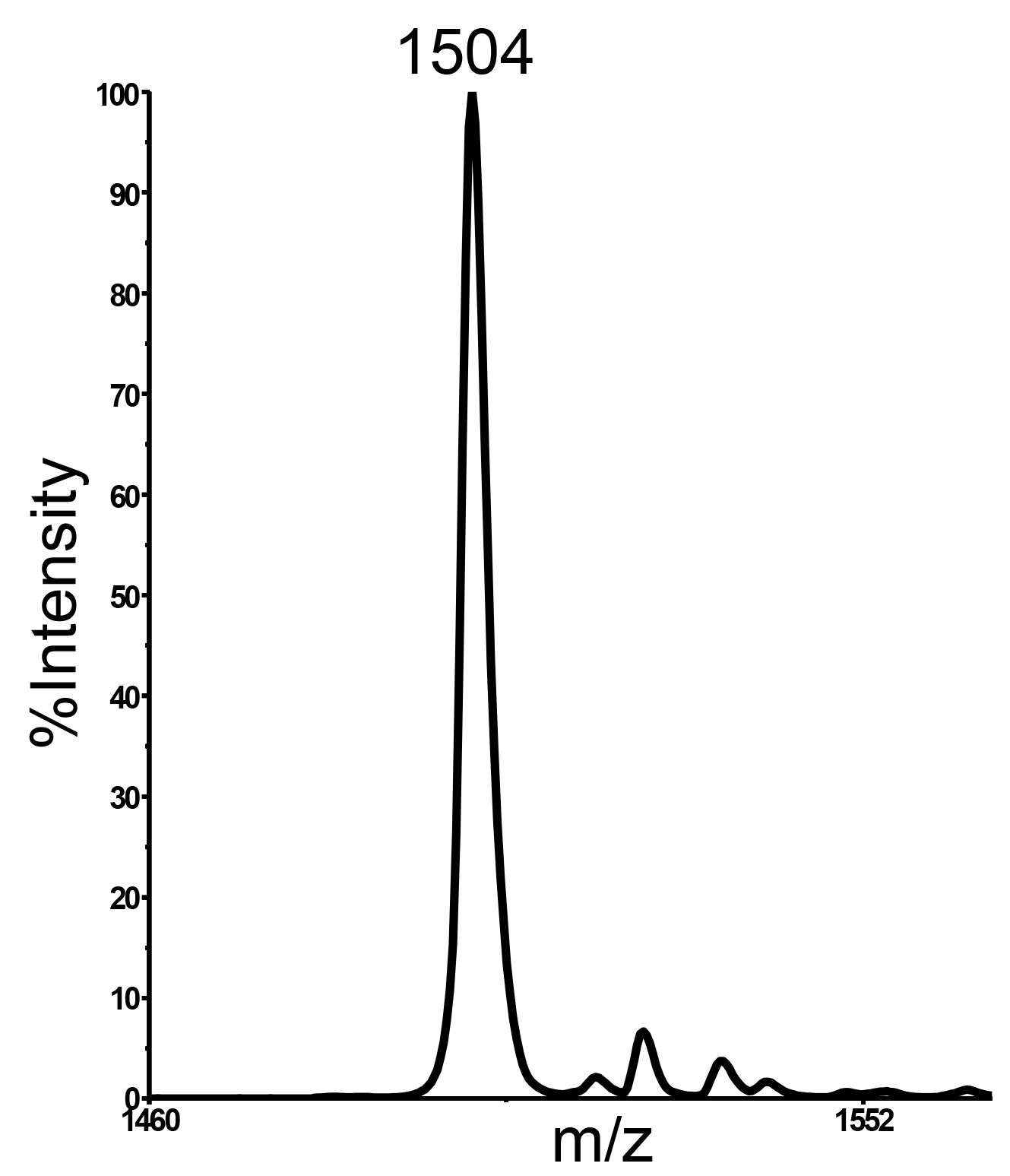


**Supplementary Figure 3.** MALDI-TOF MS data of HPLC purified BreB.


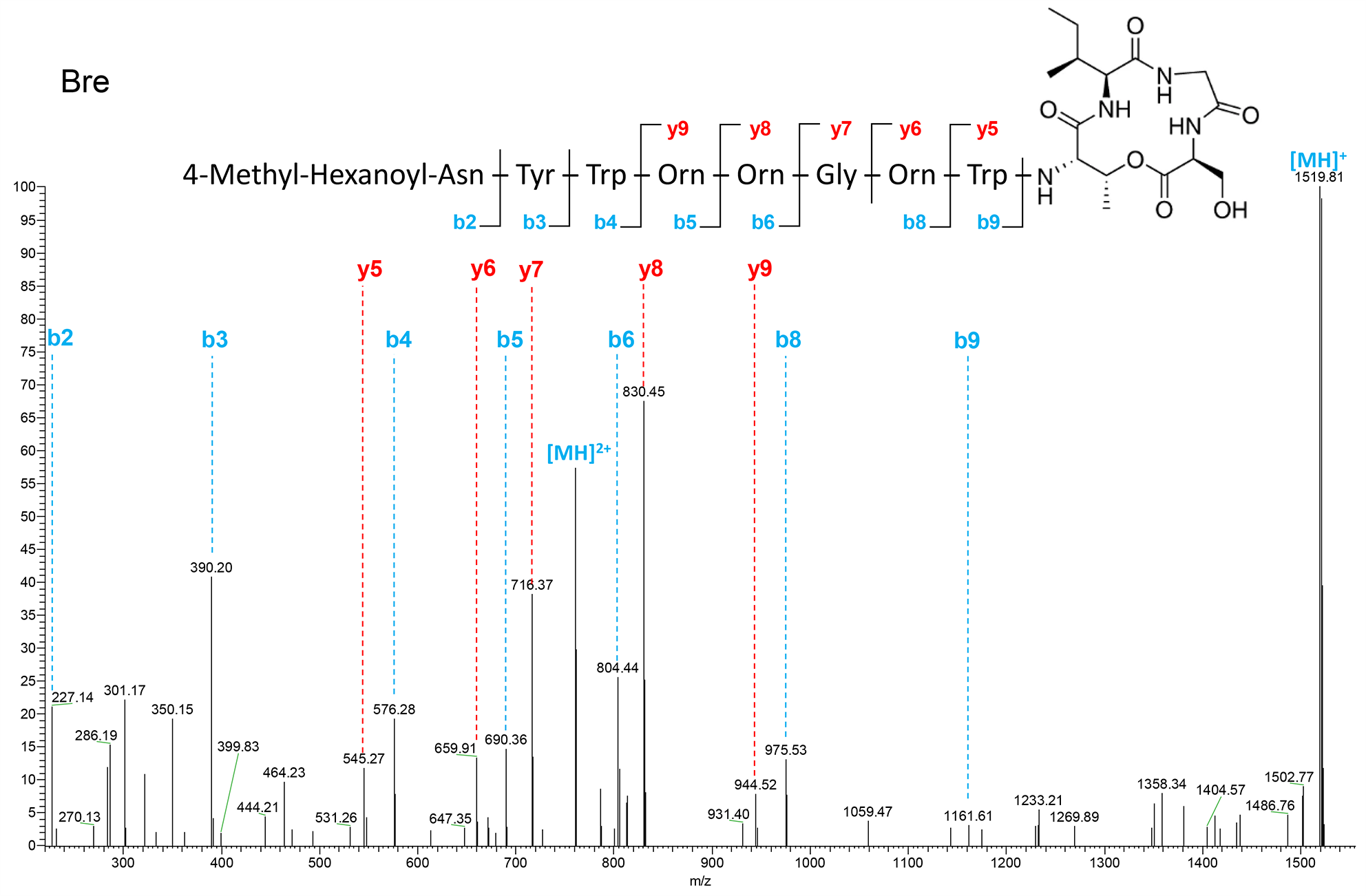


**Supplementary Figure 4.** LC-MS/MS spectrum and the proposed structures of Bre. Fragment ions are indicated.


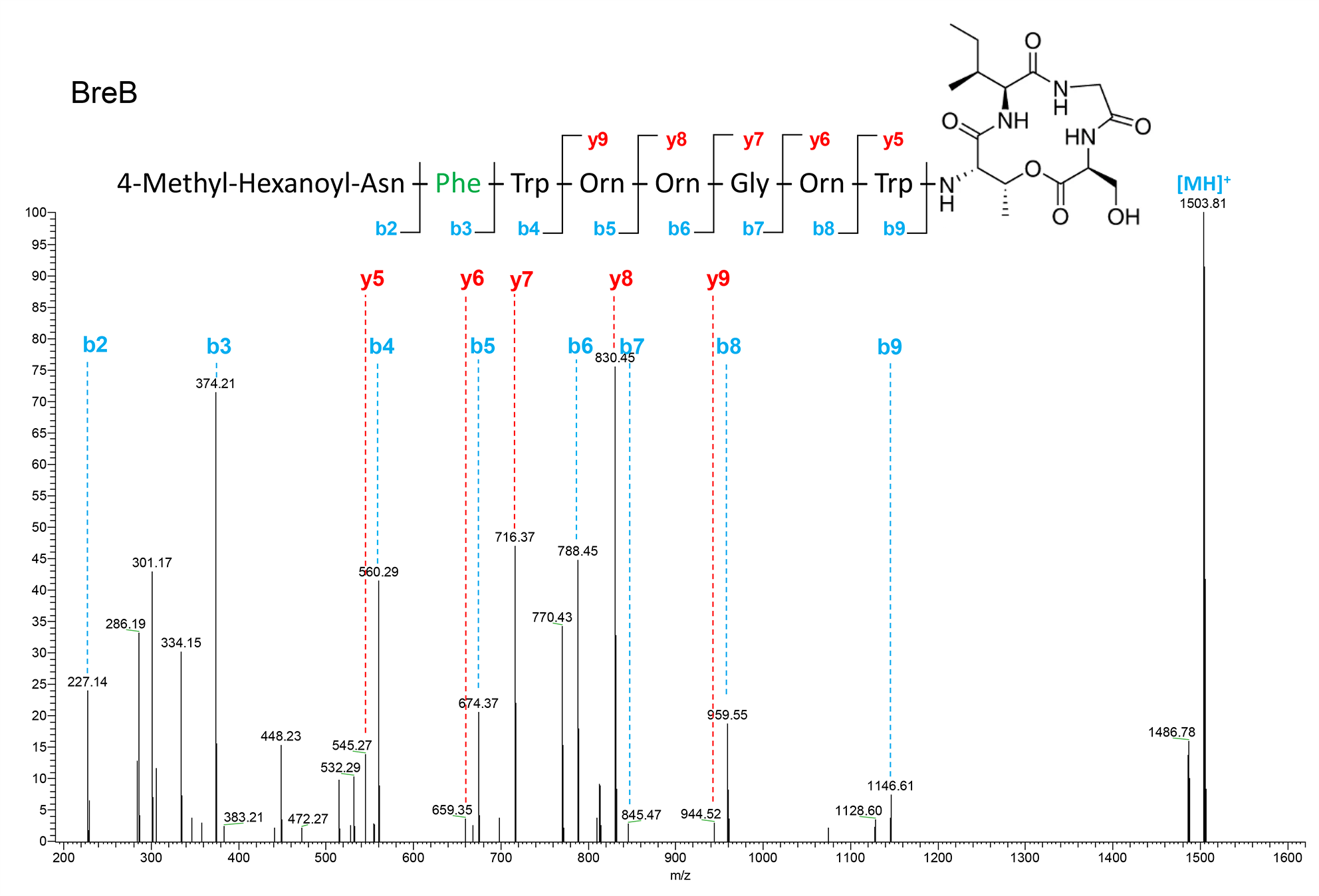


**Supplementary Figure 5.** LC-MS/MS spectrum and the proposed structures of BreB. Fragment ions are indicated.


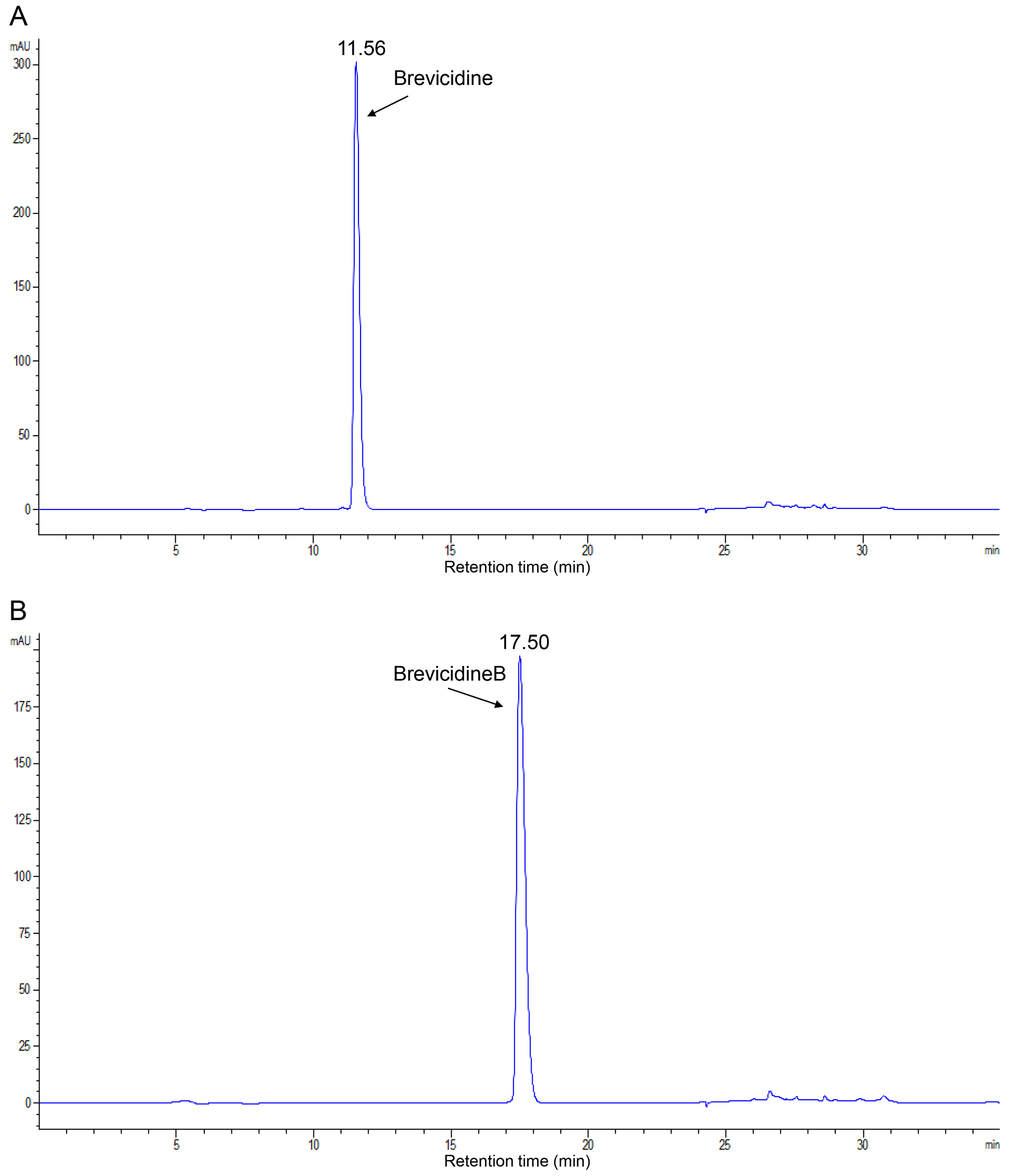


**Supplementary Figure 6.** HPLC analysis of the purities of the purified Bre and BreB. A single peak was observed for both purified Bre (**A**) and BreB (**B**), showing the high purities of these two purified peptides. Due to the more hydrophobic amino acid residue substitution, BreB showed a longer retention time on HPLC. HPLC analysis was performed on an Agilent 1260 Infinity HPLC system with a Phenomenex Aeris™ C18 column (250 × 4.6 mm, 3.6 μm particle size, 100 Å pore size). Acetonitrile was used as the mobile phase, and a gradient of 25-35% aq. MeCN over 35 min at 1 mL per min was used for separation (0-1 min, 25%-25% aq. MeCN; 1-1.01 min, 25%-30.5% aq. MeCN; 1.01-18 min, 30.5%-35% aq. MeCN; 18-20 min, 35%-95% aq. MeCN; 20-25 min, 95%-95% aq. MeCN; 25-28 min, 95%-25% aq. MeCN; 28-35 min, 25%-25% aq. MeCN). Solvent A, Milli-Q water with 0.1% TFA; solvent B, acetonitrile with 0.1% TFA.

**Supplementary Table 1** Strains used in this study.

| Strains | Characteristics and purpose |
| --- | --- |
| *Brevibacillus laterosporus* | DSM 25, host strain for production of brevicidines |
| *Bacillus cereus* | ATCC14579, indicator strain |
| *Enterococcus faecalis* | LMG16216 (VRE), indicator strain |
| *Staphylococcus aureus* | ATCC15975 (MRSA), indicator strain |
| *Enterococcus faecium* | LMG16003 (VRE), indicator strain |
| *Acinetobacter baumannii* | ATCC17978, indicator strain |
| *Escherichia coli* | ATCC25922, indicator strain |
| *Klebsiella pneumoniae* | LMG20218, indicator strain |
| *Enterobacter cloacae* | LMG02783, indicator strain |
| *Pseudomonas aeruginosa* | PAO1, indicator strain |
| *Pseudomonas aeruginosa* | LMG6395, indicator strain |
